# WWOX Phosphorylation, Signaling, and Role in Neurodegeneration

**DOI:** 10.3389/fnins.2018.00563

**Published:** 2018-08-15

**Authors:** Chan-Chuan Liu, Pei-Chuan Ho, I.-Ting Lee, Yu-An Chen, Chun-Hsien Chu, Chih-Chuan Teng, Sheng-Nan Wu, Chun-I. Sze, Ming-Fu Chiang, Nan-Shan Chang

**Affiliations:** ^1^Department of Cell Biology and Anatomy, National Cheng Kung University College of Medicine, Tainan, Taiwan; ^2^Institute of Basic Medical Sciences, National Cheng Kung University College of Medicine, Tainan, Taiwan; ^3^Institute of Molecular Medicine, National Cheng Kung University College of Medicine, Tainan, Taiwan; ^4^Graduate Institute of Medical Genomics and Proteomics, College of Medicine, National Taiwan University, Taipei, Taiwan; ^5^Department of Nursing, Chronic Diseases and Health Promotion Research Center, Chang Gung University of Science and Technology, Chiayi, Taiwan; ^6^Department of Physiology, National Cheng Kung University College of Medicine, Tainan, Taiwan; ^7^Department of Neurosurgery, Mackay Memorial Hospital, Mackay Medicine, Nursing and Management College, Graduate Institute of Injury Prevention and Control, Taipei Medical University, Taipei, Taiwan; ^8^Department of Neurochemistry, New York State Institute for Basic Research in Developmental Disabilities, New York, NY, United States; ^9^Graduate Institute of Biomedical Sciences, College of Medicine, China Medical University, Taichung, Taiwan

**Keywords:** WWOX, TRAPPC6AΔ, TIAF1, 17β-estradiol, sex steroid hormone receptor, neurodegeneration, Zfra

## Abstract

Homozygous null mutation of tumor suppressor *WWOX/Wwox* gene leads to severe neural diseases, metabolic disorders and early death in the newborns of humans, mice and rats. WWOX is frequently downregulated in the hippocampi of patients with Alzheimer’s disease (AD). *In vitro* analysis revealed that knockdown of WWOX protein in neuroblastoma cells results in aggregation of TRAPPC6AΔ, TIAF1, amyloid β, and Tau in a sequential manner. Indeed, TRAPPC6AΔ and TIAF1, but not tau and amyloid β, aggregates are present in the brains of healthy mid-aged individuals. It is reasonable to assume that very slow activation of a protein aggregation cascade starts sequentially with TRAPPC6AΔ and TIAF1 aggregation at mid-ages, then caspase activation and APP de-phosphorylation and degradation, and final accumulation of amyloid β and Tau aggregates in the brains at greater than 70 years old. WWOX binds Tau-hyperphosphorylating enzymes (e.g., GSK-3β) and blocks their functions, thereby supporting neuronal survival and differentiation. As a neuronal protective hormone, 17β-estradiol (E2) binds WWOX at an NSYK motif in the *C*-terminal SDR (short-chain alcohol dehydrogenase/reductase) domain. In this review, we discuss how WWOX and E2 block protein aggregation during neurodegeneration, and how a 31-amino-acid zinc finger-like Zfra peptide restores memory loss in mice.

## Introduction

Human and mouse WW domain-containing oxidoreductase, designated WWOX, FOR, or WOX1, has been generally regarded as a tumor suppressor since its discovery in 2000 (Bednarek et al., 2000; Ried et al., 2000; Chang et al., 2001). Human *WWOX* gene is located on a common fragile site *FRA16D*, spanning 1.1 million bases on chromosome ch16q23.3-24.1. *WWOX* is composed of 9 exons with an open reading frame (ORF) of 1245 base pairs long, encoding a 414-amino-acid (46 kDa) protein. *WWOX* gene possesses multiple long non-coding RNA *PARTICLE* (Gene *PARTICL*- ‘*P*romoter of *MAT2A*-*A*ntisense *R*adia*T*ion *I*nduced *C*irculating *L*ncRNA) triplex clusters, suggesting its control of gene expression in the genome (O’Leary et al., 2017). WWOX protein possesses two *N*-terminal WW domains, a nuclear localization signal between the WW domains, a *C*-terminal short-chain alcohol dehydrogenase/reductase (SDR) domain, and a proapoptotic *C*-terminal tail termed D3 (Bednarek et al., 2000; Ried et al., 2000; Chang et al., 2001; Hong et al., 2007; Lin et al., 2011; Abu-Remaileh and Aqeilan, 2015, Abu-Remaileh et al., 2015; Huang and Chang, 2018).

The WW domain participates in protein/protein interactions for transducing signals (Chang et al., 2007; McDonald et al., 2012; Reuven et al., 2015). The first WW domain of WWOX binds PPxY or PPPY-containing proteins (e.g., p73, ErbB-4, SIMPLE, WWBP1, WWBP2, Ezrin, AP-2g, Runx-2, and many others) (Ludes-Meyers et al., 2004; Jin et al., 2006; Chang et al., 2007; McDonald et al., 2012; Reuven et al., 2015). When Tyr33 in the first WW domain is phosphorylated, activated WWOX acquires an enhanced capability in binding a broad spectrum of proteins (Chang et al., 2007; Reuven et al., 2015), including p53 (Chang et al., 2001, 2003a,b, 2005a,b, 2007), c-Jun *N*-terminal kinase (JNK) (Chang et al., 2003a), Zinc finger-like protein that regulates apoptosis (Zfra) (Hong et al., 2007), c-Jun and cAMP response element binding protein (CREB) (Li et al., 2009) and others. The second tandem WW domain assists synergistically with the first WW domain in enhancing the protein/protein binding (Farooq, 2015). Transiently overexpressed WWOX frequently sequesters transcription factors in the cytoplasm, and thereby blocks their transcription for prosurvival proteins in the nucleus in cancer cells *in vitro* (Gaudio et al., 2006). In contrast, endogenous WWOX binds and co-translates with many transcription factors to relocate to the nucleus to enhance or block neuronal survival under sciatic nerve dissection (Li et al., 2009). Endogenous trafficking protein particle complex 6A (TRAPPC6A) acts as a carrier for WWOX to undergo nuclear translocation (Chang et al., 2015). Indeed, WWOX works together with many transcription factors to either support neuronal survival or death under physiological or pathological conditions.

## Tyr33-Phosphorylated WWOX in Apoptosis and in Temperature-Related Bubbling Cell Death (BCD)

The proapoptotic function of WWOX has been previously reviewed (Chang, 2002, 2015; Chang et al., 2003b, 2007; Huang and Chang, 2018). Briefly, UV irradiation activates cytosolic WWOX via Tyr33 phosphorylation (pY33-WWOX), followed by binding Ser46-phosphorylated p53. Both proteins relocate to the mitochondria or nuclei to induce cell death (Chang et al., 2003a, 2005a). As an inhibitor, JNK or Zfra suppresses WWOX in inducing apoptosis (Chang et al., 2003a; Hong et al., 2007; Aderca et al., 2008). Zfra acts by reducing Tyr33 phosphorylation in WWOX (Hong et al., 2007). Zfra binds the *N*-terminal WW domain and *C*-terminal SDR domain of WWOX. This binding interferes with Tyr33 phosphorylation by tyrosine kinase Src (Aqeilan et al., 2004).

At temperatures lower than 37°C, WWOX is needed for a recently described type of cell death, designated bubbling cell death (Chen et al., 2015; Chang, 2016). BCD is not apoptosis, necroptosis, or necrosis. When cells are subjected to UV irradiation and cold shock followed by culturing at 37°C, the cells undergo apoptosis (e.g., caspase activation, whole cell and nuclear condensation, DNA fragmentation, etc.). However, if the UV/cold shock-treated cells are incubated at a lower temperature (e.g., 4, 10, or 22°C), they generate, in most cases, a nuclear nitric oxide (NO)-containing bubble per cell. Some cells may generate 2–3 bubbles. The bubble continues to inflate and finally is released from the cell membrane. The cells die later on. Membrane phosphatidylserine flip over, caspase activation and DNA fragmentation, which are found in apoptosis, are not involved in BCD. Raising the temperature back to 37°C resumes the event to apoptosis. If cells are devoid of WWOX (e.g., *Wwox*^-/-^ MEF), cell death is retarded (Chen et al., 2015; Chang, 2016). Overall, UV energy is absorbed by the nucleus, and cold shock assists the rapid relocation of cytosolic p53, WWOX, and NOS2 to the nucleus. Nitric oxide synthase NOS2 is responsible for the bubble generation that leads to cell death (Chen et al., 2015; Chang, 2016).

## Activated WWOX Induces Cell Death From the Mitochondria and Nuclei

### WWOX in Neuronal Injury

Constant light-induced retinal neural degeneration involves WWOX activation and pY33-WWOX accumulation in the mitochondria and nuclei to cause damage and death (Chen et al., 2005). Neurotoxin MPP^+^ (1-methyl-4-phenylpyridinium) also induces pY33-WWOX upregulation and nuclear accumulation to cause neuronal death in rats (Lo et al., 2008). During the acute phase of sciatic nerve dissection, pY33-WWOX, along with its interacting transcription factors, becomes accumulated in the nucleus that leads to the rapid death of the large-sized neurons *in vivo* (Li et al., 2009). WWOX blocks the prosurvival function of CREB-, CRE-, and AP-1-mediated promoter activation *in vitro* (Li et al., 2009). In stark contrast, WWOX enhances the promoter activation governed by c-Jun, Elk-1 and NF-κB (Li et al., 2009). Apparently, a balance in the protein levels for WWOX and transcription factors is critical in determining the fate of dissected neurons.

### Hyal-2/WWOX Signaling in Traumatic Brain Injury (TBI) Links to BCD

WWOX localizes in many subcellular compartments, including cell membrane, mitochondrion, lysosome, nucleus, and others. WWOX does not have a membrane localization signal. It is anchored, in part, to the membrane/cytoskeleton area by hyaluronidase Hyal-2 (Hsu et al., 2009, 2016, 2017) and Ezrin (Jin et al., 2006). WWOX acts as a transducer in many stress-related signal pathways induced by tumor necrosis factor (TNF), chemotherapeutic drugs, UV irradiation (Chang et al., 2007, 2010, 2014, 2015; Abu-Remaileh and Aqeilan, 2015), Wnt/β-catenin (Bouteille et al., 2009), transforming growth factor-β (TGF-β) (Hsu et al., 2009; Chang et al., 2010), complement C1q (Hong et al., 2009), hyaluronan and Hyal-2 (Chang et al., 2010; Hsu et al., 2016, 2017), sex steroid hormones (Su et al., 2012), T cell differentiation reagents (Huang et al., 2016), and others.

**FIGURE 1:**
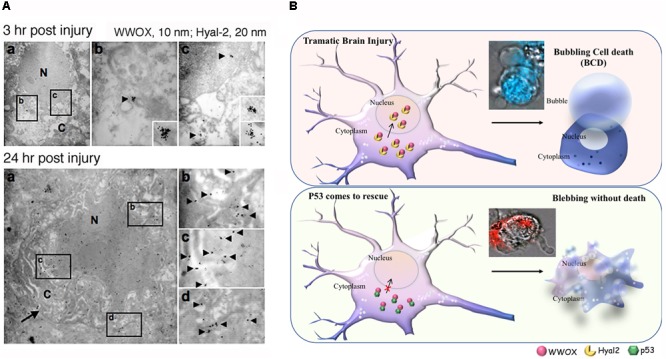
Potential role of WWOX and BCD in neuronal death during traumatic injury. **(A)** Needle insult to the brain was carried out in rats. Post injury for 3 and 24 h, the animals were sacrificed. By immunoelectron microscopy, accumulation of Hyal-2 and WWOX is found in the nuclei of dying neurons in the brain cortex (Hsu et al., 2017). **(B)** Nuclear accumulation of Hyal-2 and WWOX leads to BCD (Chen et al., 2015). Both schematic graphs and a real-time image are shown. If p53 competes with Hyal-2 to complex with WWOX, both p53/WWOX proteins are retained in the cytoplasm and the extent of Hyal-2/WWOX complex is reduced, no BCD occurs (Hsu et al., 2017). (Data in **A** is adapted from Hsu et al., 2017, republishing according to the guideline of *Oncotarget*).

During TBI, activation of the Hyal-2/WWOX/Smad4 signaling complex causes neuronal death (Hsu et al., 2017) (**Figure 1A**). Hyal-2 and WWOX are accumulated in the nuclei of damaged neurons in rat brain (Hsu et al., 2017) (**Figure 1A**). Hyal-2 is a cognate receptor for hyaluronan and TGF-β1 (Hsu et al., 2009, 2016, 2017). Both hyaluronan and TGF-β1 may utilize the Hyal-2/WWOX/Smad4 signaling to enhance the cell survival or death. It has been shown that long-term overexpression of TGF-β1 causes neurodegeneration in mice (Ueberham et al., 2005).

Bubbling cell death can also occur at 37°C. For example, when cells are transiently overexpressed with hyaluronidase Hyal-2 and WWOX followed by treating with high-molecular-weight hyaluronan, BCD occurs at 37°C (Hsu et al., 2017) (**Figure 1B**). Hyaluronan binds membrane Hyal-2 to initiate the Hyal-2/WWOX signaling, and that both Hyal-2 and WWOX are accumulated in the nuclei. It is reasonable to assume that during TBI, the nuclear Hyal-2 and WWOX may exert BCD due to the production of NO. Formation of nuclear bubbles in the dying neurons *in vivo* is unknown. However, bubble formation *in vivo* is difficult to detect, because it is technically impossible to fix bubbles for microscopic examination. Reactive oxygens species (ROS) are rapidly upregulated during TBI (Bains and Hall, 2012). WWOX, via its *C*-terminal SDR domain, controls the generation of ROS in *Drosophila* (O’Keefe et al., 2011) and mammalian cells (Dayan et al., 2013). Also, the SDR domain of WWOX controls the cellular outgrowths caused by genetic deficiencies of the components of the mitochondrial respiratory complexes in *Drosophila* (Choo et al., 2015). Under physiologic conditions, oxidative phosphorylation sustains WWOX expression (Dayan et al., 2013). However, when glycolysis (or Warburg metabolism) goes up in aberrant cells, WWOX expression is downregulated (Dayan et al., 2013). Reduced WWOX levels in *Drosophila* allow cellular outgrowths to various extent caused by genetic deficiencies of components of the mitochondrial respiratory complexes and aberrant ROS production (Choo et al., 2015). Together, WWOX participates in TBI and this is related with ROS generation and brain tissue repair.

## WWOX in Neurodegeneration *In Vivo*

### Pathological Features in Neurodegeneration

Neurodegenerative diseases (NDs) encompass a heterogeneous group of chronic progressive diseases, each affecting specific central nervous system (CNS) compartment. The pathologies of NDs are not specific for each individual disease. Neurofibrillary tangles and Lewy bodies, for example, may appear in non-demented and non-idiopathic Parkinson disease patients. Also, the pathological or clinical features may overlap. Over the past decades, many animal models have been established to seek potential propagation mechanisms and associated risk factors for NDs (Martin, 2012; Niccoli and Partridge, 2012; Dugger and Dickson, 2016; Hartl, 2017; Chételat, 2018). Aging-related stress, oxidative stress, reduced mitochondrial function, altered subcellular transport, and activation of the ER stress and unfolded protein response (UPR) pathways are considered important during neurodegeneration (Martin, 2012; Hartl, 2017).

In the aging processes, chaperones may become dysregulated and the degradation machineries stop working properly, which leads to protein misfolding, aggregation, and accumulation for neuronal damage. Among these, UPR exists in the mitochondria and the endoplasmic reticulum, along with disordered cytosolic heat shock response, ubiquitin-proteasome system, and autophagy (Taylor and Dillin, 2013; Hartl, 2017). Presence of aberrant protein aggregates, inclusion bodies and/or tangled fibrous proteins in the aging neurons, glial cells, and brain matrix is the pathological hallmarks of neurodegeneration (Ross and Poirier, 2005; Richter-Landsberg and Leyk, 2013; Higuchi-Sanabria et al., 2018). Furthermore, formation and spread of prion-like Aβ aggregates occur during AD progression, and this is not due to overexpression of APP (amyloid precursor protein) (Ruiz-Riquelme et al., 2018). Prion protein in the exosomes facilitates the spreading and aggregation of neurotoxic Aβ (Hartmann et al., 2017).

### WWOX Deficiency Leads to Severe Neural Damage and Metabolic Disorders

The WWOX protein is heterogeneously expressed in the central nervous system. WWOX-positive stains are found in the human cerebrum, specifically in the pyramidal neurons and astrocytes from the frontal and occipital cortices, and in the nucleus caudate, pons and nuclei olivaris of medulla. Neuropils and small neurons are also immunoreactive to WWOX antibody. However, parietal, limbic and temporal cortices and substantia nigra are minimal or negative for WWOX immunoreactivity (Nunez et al., 2005). In the developing mouse brain, WWOX protein expression is essentially present in every brain region and the expression level is reduced in the newborns (Chen et al., 2004). In the adult brain, WWOX is abundant in the epithelial cells of the choroid plexus and ependymal cells, while a low to moderate level of WWOX is observed within white matter tracts, such as axonal profiles of the corpus callosum, striatum, optic tract, and cerebral peduncle (Chen et al., 2004).

Despite its role in cell death, WWOX is essential in homeostasis *in vivo*. *WWOX*/*Wwox gene* deficiency severely affects normal physiological functions, especially in embryonic neural development (Chen et al., 2004; Aldaz et al., 2014; Chang et al., 2014, 2015; Tabarki et al., 2015). Deficiency of *WWOX*/*Wwox* gene due to point mutations or homozygous nonsense mutation may result in childhood onset autosomal recessive cerebellar ataxia and epilepsy, growth retardation, microcephaly with seizure, retinal degeneration, and early death at 16 months of age (Valduga et al., 2015; Alkhateeb et al., 2016; Elsaadany et al., 2016). Similar observations are shown in rats (Suzuki et al., 2009).

*WWOX* gene is involved in the regulation of lipid homeostasis and metabolism (Ludes-Meyers et al., 2004, 2009; Lee et al., 2008; Yang et al., 2012; Dayan et al., 2013; Iatan et al., 2014; Li et al., 2014; Abu-Remaileh and Aqeilan, 2015; Abu-Remaileh et al., 2015). *WWOX* gene alteration is associated with the low plasma high-density lipoprotein cholesterol (HDL-C) levels and aberrant HDL-C and triglyceride levels (Lee et al., 2008; Sáez et al., 2010). Furthermore, whole body and liver conditional *Wwox* knockout mice revealed a significant role for *Wwox* in regulating HDL and lipid metabolism (Iatan et al., 2014).

Interference in lipid metabolism may be a critical contributor in the pathogenesis of neurological diseases. For example, WWOX is not expressed in the lipid-rich myelin sheath in the normal neurons, but activated pY33-WWOX is accumulated in the myelin sheath during neurotoxin MPP^+^-induced neuronal death (Lo et al., 2008). While both apolipoprotein E (Apo E) and WWOX are involved in AD and TBI, the functional relationship between these two proteins (e.g., binding) needs further elucidation. Taken together, WWOX plays a crucial role in neural development and lipid metabolism. Without WWOX, severe neural diseases, metabolic disorders and early death occur in humans and animals.

### *WWOX* Gene Expression in the Brain

**FIGURE 2:**
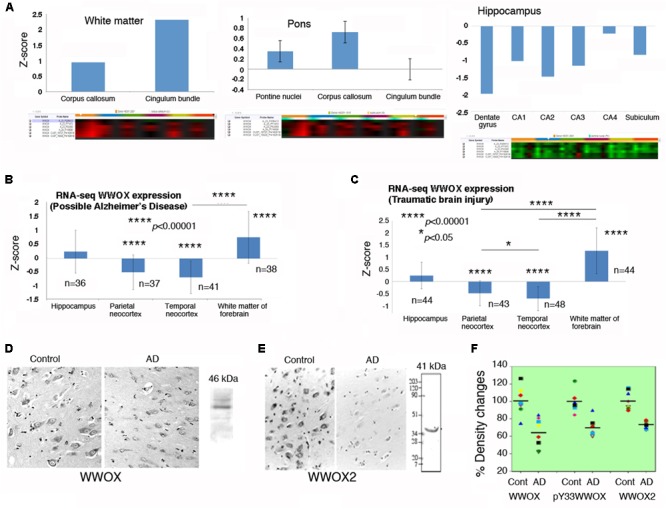
*WWOX* gene and protein expression in human brain. **(A)** *WWOX* gene expression was analyzed using the database in the Allen Brain Atlas (http://www.brain-map.org). Detectable signals for *WWOX* gene expression were found in six postmortem normal individuals (age 42.5 ± 13.4; 3 Caucasians, 2 blacks, 1 Hispanic). Representative *WWOX* gene expression levels in the brain white matter, pons and hippocampus are shown. Also, see the **Supplementary Table S1** for *WWOX* gene expression in the normal brains (around one-fold changes for all indicated regions). **(B,C)** In the “Possible AD” and “Traumatic Brain Injury” groups (77–100+ years old), *WWOX* gene expression levels are shown in the indicated brain areas. **(D,E)** Expression of wild type WWOX (46 kDa) and isoform WWOX2 (41 kDa) is downregulated in the neurons of AD hippocampi compared with normal controls (a representative set from five immunostains; magnification, 200×; data from Sze et al., 2004). **(F)** In AD patients, the protein levels for WWOX (*n* = 8), isoform WWOX2 (*n* = 8), and pY33-WWOX (*n* = 6) are significantly downregulated in the hippocampi as determined by Western blotting, compared to age-matched controls (∼32 ± 5% reduction, *p* < 0.005; data with minor revisions for the art work are adapted from Sze et al., 2004; republishing according to the guideline of the *Journal of Biological Chemistry*).

By analyzing the database in the Allen Brain Atlas^1^, *WWOX* gene expression levels are shown to be significantly downregulated in the postmortem normal hippocampus, compared to those in the pons and white matter (*n* = 6; age 42.5 ± 13.4; 3 Caucasians, 2 blacks, 1 Hispanic) (**Figure 2A**). There were only six normal brain samples exhibiting detectable signals for *WWOX* gene expression (as shown in the **Supplementary Table S1**). *WWOX* gene expression is upregulated in the cingulum bundle of the white matter by 2.31-fold, and the central glial substance of the myelencephalon by 2.78-fold.

In the “Possible AD” group (77 to 100+ years old), *WWOX* gene expression levels are barely changed in the hippocampus (**Figure 2B**). Also, compared to the hippocampus, *WWOX* gene expression is significantly downregulated in the parietal and temporal neocortex, but is significantly upregulated in the white matter of the forebrain (**Figure 2B**). Interestingly, similar expression profiles are observed in the “Traumatic Brain Injury (TBI)” group (77 to 100+ years old; **Figure 2C**).

Also, in other gene databases (GTEx, Illumina, BioGPS, and CGAP SAGE, as summarized in the GeneCard^2^), *WWOX* gene expression levels in the brain, cerebellum, cortex, spinal cord and tibial nerve are similar to those from other tissues and organs in normal humans. However, WWOX protein expression levels are significantly increased in the human fetal brains (GeneCard database shown above). This is in agreement with our observations using mouse fetal brains (Chen et al., 2004).

### WWOX Protein Downregulation in Alzheimer’s Disease (AD)

It is generally agreed that gene expression cannot always correlate with protein expression. The aforementioned *WWOX* gene expression levels do not correlate positively with the extent of WWOX protein expression. For example, downregulation of *WWOX* gene occurs in the hippocampi of young adults (**Figure 2A**) and many other areas (**Supplementary Table S1**). However, WWOX protein expression levels are detectable in neurons of many regions in the brain (Nunez et al., 2005).

Indeed, significant downregulation of the protein level for WWOX, isoform WWOX2, and pY33-WWOX has been shown in the hippocampi of AD patients, compared to age-matched controls (Sze et al., 2004) (**Figures 2D–F**). However, during sciatic nerve injury, rapid upregulation of *Wwox* gene expression occurs in less than 30 min in the neurons of dorsal root ganglion, followed by significant upregulation of WWOX protein and its Tyr33 phosphorylation in the damaged neurons in 24 h (Li et al., 2009). Activated WWOX is needed to initiate neuronal death in the damaged tissue.

There is no positive correlation between *WWOX*/*Wwox* mRNA expression and protein expression. For example, translational blockade of *Wwox* mRNA has been shown in the development of skin squamous cell carcinoma (SCC) in hairless mice (Lai et al., 2005). During the acute exposure of hairless mice to UVB, both WWOX and pY33-WWOX proteins are upregulated in epidermal cells in 24 h. SCCs then start to develop in 3 months. There are significant reductions in WWOX and pY33-WWOX proteins in the SCC cells. However, no downregulation of *Wwox* mRNA occurs (Lai et al., 2005). In SCC patients, significant reduction of WWOX and pY33-WWOX proteins are observed in non-metastatic and metastatic cutaneous SCCs, whereas no downregulation of *WWOX* mRNA occurs (Lai et al., 2005). Together, *WWOX/Wwox* mRNA is subjected to translational blockade in the skin and probably other tissues and organs under pathological conditions.

### WWOX Control of Neuronal Survival via Binding and Suppressing Tau and Tau Hyperphosphorylating Enzymes

**FIGURE 3:**
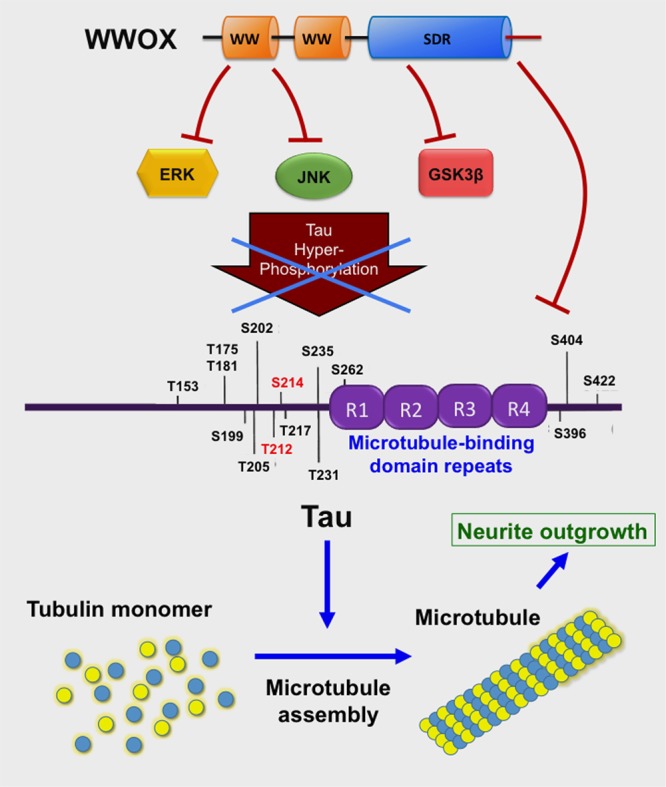
WWOX limits Tau hyperphosphorylation and aggregation. The *C*-terminal SDR domain of WWOX physically binds GSK3β preventing hyperphosphorylation of Tau (Sze et al., 2004; Wang et al., 2012). Also, the first WW domain binds JNK, thereby preventing Tau hyperphosphorylation (Sze et al., 2004). The first WW domain binds ERK (Huang et al., 2016). Tau protein supports polymerization of tubulin monomers to assemble microtubules, which are needed for neurite outgrowth (Wang et al., 2012).

Compelling evidence reveals that WWOX is likely to slow down neurodegeneration such as in AD. For example, the *C*-terminal SDR domain of WWOX binds and limits the enzymatic activity of glycogen synthase kinase 3β (GSK-3β) (Sze et al., 2004; Wang et al., 2012) (**Figure 3**). GSK-3β is known to hyperphosphorylate Tau which leads to restriction of neurite outgrowth, and prevention of neuronal differentiation resulting in formation of neurofibrillary tangles (NFTs) and senile plaques (SPs) in AD (Augustinack et al., 2002; Avila et al., 2010; Chang et al., 2014; Llorens-Martin et al., 2014; Sze et al., 2004). WWOX binds Tau via its SDR domain (Sze et al., 2004) (**Figure 3**), and prevents enzyme-dependent Tau hyperphosphorylation (Sze et al., 2004). WWOX contains two potential GSK-3β-binding FXXXLI/VXRLE motifs (Wang et al., 2012). Precisely, the α-helical segment (amino acids #388 to 407) at the SDR domain of WWOX interacts physically with the GSK3β binding-pocket *α*-helix (amino acid #262 to 273) (Wang et al., 2012). Transiently overexpressed WWOX inhibits GSK-3β-stimulated S396 and S404 phosphorylation within the microtubule domains of Tau. Consequently, WWOX represses GSK-3β activity in hyperphosphorylating tau, restores tau’s ability to assemble the microtubule network, and promotes neurite outgrowth in neuroblastoma SH-SY5Y cells. When WWOX is knocked down by small interfering RNA, retinoic acid is not able to mediate differentiation of SH-SY5Y cells (e.g., neurite outgrowth) (Wang et al., 2012).

The WWOX also binds JNK via its Tyr33-phosphorylated first WW domain, and the binding results in neutralization of the functions of both proteins in a reciprocal manner (Chang et al., 2003a) (**Figure 3**). Additionally, the first WW domain of WWOX physically interacts with ERK (extracellular signal-regulated kinase) (Huang and Chang, 2018). ERK has been implicated in Tau hyperphosphorylation (Augustinack et al., 2002) (**Figure 3**). Cyclin dependent kinase 5 (Cdk5) hyperphosphorylates many substrates such as amyloid precursor protein, tau and many other proteins in the brain (Shah and Lahiri, 2015); however, functional interaction between WWOX and CDK5 has never been documented.

### TIAF1 and TRAPPC6AΔ Protein Aggregates in the Hippocampi of Mid-Aged Normal Individuals

**FIGURE 4:**
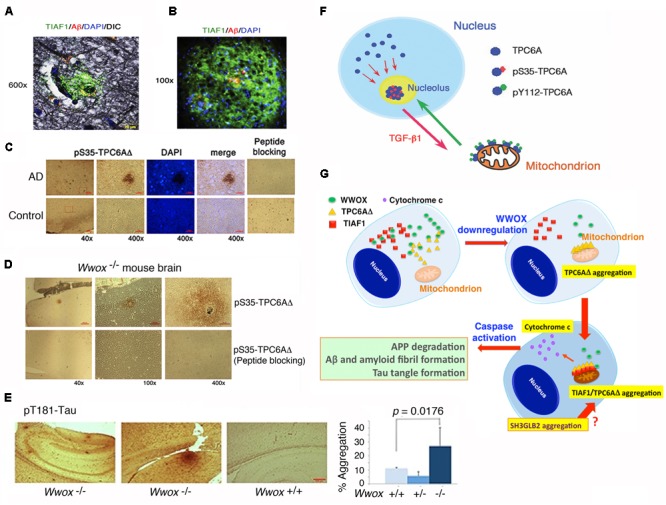
TPC6AΔ and TIAF1 in a cascade of protein aggregation and WWOX blocks the aggregation. **(A)** Representative human AD hippocampal tissue sections were pre-stained with Bielschowsky stain, followed by staining with specific antibody against TIAF1 (green), and Aβ (red) and DAPI for nuclei. A representative confocal image of a plaque is shown (Lee et al., 2010). **(B)** Shown is a TIAF1-containing plaque from a hippocampal section of a 9-month-old APP/PS1 transgenic mouse, containing Aβ aggregates in the center (Lee et al., 2010). **(C)** In representative human brain cortical tissue sections from AD patients and age-matched controls, a pS35-TPC6AΔ-containing plaque is shown. In negative controls, the immunizing peptide blocks the immunoreactivity (Chang et al., 2015). **(D,E)** Presence of pS35-TPC6AΔ and pT181-Tau aggregates is shown in the cortex and hippocampus of 3-week-old *Wwox* knockout mice (Chang et al., 2015). **(F)** Endogenous TPC6A and TPC6AΔ shuttle between nucleoli and mitochondria. Ser35 phosphorylation supports shuttling from the nucleus to the nucleolus, and Tyr112 phosphorylation is needed for translocation from the nucleolus to the mitochondrion (Chang and Chang, 2015). **(G)** Upon WWOX downregulation, a sequential protein aggregation cascade occurs. When WWOX level is reduced, pS35-TPC6AΔ starts to polymerize and recruit pS37-TIAF1 for further polymerization and accumulation in the outer membrane of mitochondria (Chang and Chang, 2015; Chang et al., 2015). The aggregated pS35-TPC6AΔ and pS37-TIAF1 cause caspase 3 activation and cytochrome c release. The activated caspase 3 leads to APP degradation and formation of Aβ and amyloid fibrils and Tau tangles. SH3GLB2 aggregation (Lee et al., 2017) occurs probably right after that of pS37-TIAF1. (All data are adapted with revisions in art work from Lee et al., 2010; Chang and Chang, 2015; Chang et al., 2015, under the guidelines of the publishers).

In an inducible transgenic mouse model, neuron-specific expression of TGF-β in the neocortex, hippocampus and striatum for a long term results in deposition of amyloid fibrils in these brain areas (Ueberham et al., 2005). Deposits of apolipoprotein E (ApoE) are also found in perivascular areas (Ueberham et al., 2005). When TGF-β induction stops, the amyloid and ApoE aggregates stably remain in the brain and vascular lesions. We have discovered a few novel proteins, whose aggregation is found in the brain hippocampal and cortical areas of both non-demented healthy individuals and demented AD patients. TGF-β1-induced antiapoptotic factor 1 (TIAF1; 12 kDa) is involved in the pathogenesis of AD and cancer, as well as in allograft rejection by activated T helper cells (van der Leij et al., 2003; Lee et al., 2010; Hong et al., 2013; Chang and Chang, 2015). Presence of aggregated TIAF1 protein in the dead neurons is shown in the hippocampi of middle-aged normal humans (Lee et al., 2010; Chang and Chang, 2015). Notably, little or no Aβ aggregates are found in the TIAF1 plaques in the mid-aged humans (Lee et al., 2010) (**Figure 4A**). For example, TIAF1 aggregation is detected in 59% of non-demented control hippocampi (age 59.0 ± 17.0, *n* = 41), and only 15% of the total samples have Aβ aggregates, as determined by filter retardation assay (Lee et al., 2010). However, 54% of TIAF1 aggregation is shown in the hippocampi of older postmortem AD patients (age 80.0 ± 8.8, *n* = 97), in which 48% of the total AD samples possess Aβ aggregates. Presence of a representative TIAF1-containing plaque from the hippocampus of a 9-month-old APP/PS1 transgenic mouse is shown (**Figure 4B**). A minimal amount of Aβ aggregates is found within the center of the plaque. The observations imply that TIAF1 aggregates are difficult to remove with age by the ubiquitination/proteasomal degradation system. *In vitro* analysis revealed that TIAF1 undergoes self-polymerization and this leads to amyloid β formation (Lee et al., 2010). Together, TIAF1 aggregation occurs in the middle age and this may result in slow formation of amyloid β in humans (Lee et al., 2010).

TGF-B-induced TIAF1 self-aggregation leading to the formation of Aβ aggregates probably occurs via a signaling pathway independently of the type II TGF-β receptor (Lee et al., 2010; Hong et al., 2013; Chang and Chang, 2015). TGF-β binds membrane Hyal-2 to initiate a non-canonical Hyal-2/WWOX/Smad4 pathway (Hsu et al., 2009, 2016, 2017). TIAF1 physically binds Smad4 and strongly suppresses SMAD-regulated promoter activation, and Smad4 blocks TIAF1 aggregation caused by TGF-β (Lee et al., 2010). In addition, p53 binds TIAF1. In the absence of TIAF1, pS15-p53 fails to undergo nuclear translocation (Schultz et al., 2004). Interestingly, without p53, self-aggregating TIAF1 spontaneously activates the SMAD-regulated promoter to cause cell death (Chang et al., 2012).

Under physiologic conditions, TGF-β1 promotes binding of TIAF1 with Smad4, and the TIAF1/Smad4 complex co-relocates to the nucleus and modulates gene transcription (Lee et al., 2010; Chang et al., 2012; Hong et al., 2013). This normal signaling event does not cause protein aggregation. However, under aberrant signaling, TGF-β1 causes TIAF1 aggregation and reduces its binding with membrane APP, thus leading to APP de-phosphorylation at Thr688 and then degradation and production of amyloid β monomer, intracellular domain of the APP intracellular domain (AICD), and amyloid fibrils (Henriques et al., 2009; Lee et al., 2010; Chang et al., 2012; Hong et al., 2013). Presence of aggregated TIAF1 in the peritumor coats of metastatic brain tumor cells does not cause cancer cell death (Lee et al., 2010; Chang et al., 2012; Hong et al., 2013). However, the coat-associated TIAF1 aggregates are cytotoxic to neurons (Lee et al., 2010).

### TRAPPC6AΔ Protein Aggregation Is Upstream of TIAF1

We have identified a TGF-β-induced trafficking protein particle complex 6A (TRAPPC6A or TPC6A) (Chang and Chang, 2015; Chang et al., 2015). *TRAPPC6A*/*Trappc6a* gene is associated with skin pigment formation in mice (Gwynn et al., 2006), AD in humans (Hamilton et al., 2011), and other neural diseases (Mohamoud et al., 2018). An intra-*N*-terminal deletion isoform of TRAPPC6A, designated TRAPPC6AΔ or TPC6AΔ, tends to spontaneously form aggregates or plaques in the extracellular matrix of the hippocampi of postmortem middle-aged normal humans and older AD patients (**Figure 4C**) and 3-week-old *Wwox* gene knockout mice (**Figure 4D**) (Chang and Chang, 2015; Chang et al., 2015). Presence of pT181-Tau, a marker for tau phosphorylation and aggregation in mice, is also shown in the cortex of *Wwox* knockout mice, but is barely detectable in the wild type and heterozygous *Wwox* mice (**Figure 4D**). Conceivably, without WWOX, cellular proteins tend to undergo aggregation.

TPC6A aggregates are also present in the human brain cortex and hippocampus, which are ∼50 and 40% positive, respectively, for both control (59 ± 17 years old; *n* = 42) and AD (80 ± 8.8 years old; *n* = 96) groups (Chang et al., 2015), suggesting that the aggregated proteins are stable and hard to undergo degradation with age. In comparison, protein aggregates for pY33-WWOX are significantly reduced by ∼40% in the AD samples, compared to non-demented controls (Chang et al., 2015). Again, compared with the non-demented controls, tangled tau and Aβ aggregates are significantly increased in the AD samples (Chang et al., 2015). If our observations hold true, TPC6AΔ/TIAF1 starts polymerization in the middle age, and takes at least 10–40 years to generate significant amounts of tau and amyloid β protein aggregates for clinically defined AD symptoms.

We have recently determined that endogenous TPC6A undergoes a novel mitochondrion-nucleolus shuttling (**Figure 4F**) (Chang and Chang, 2015). TGF-β1 causes nuclear TPC6A to undergo Ser35 phosphorylation, followed by entering the nucleoli and then relocating to the mitochondria as a dimer, which probably requires phosphorylation at Tyr112. The mitochondrial TPC6A shuttles back to the nucleolus. TPC6A carries WWOX to the nucleus.

TPC6AΔ protein possesses an internal frame deletion of amino acids #29–42 at the *N*-terminus. Wild type TPC6A is less likely to undergo aggregation. Both TPC6A and TPC6AΔ proteins are able to shuttle between nuclei and mitochondria (Chang and Chang, 2015; Chang et al., 2015). Under aberrant signaling, TPC6AΔ molecules are accumulated as aggregates in the mitochondria, where TIAF1 binds TPC6AΔ. Both proteins induce caspase activation and apoptosis (**Figure 4G**) (Chang and Chang, 2015; Chang et al., 2015). A BAR domain-containing SH3GLB2 (SH3 Domain Containing GRB2 Like, Endophilin B2) is a potential downstream protein for aggregation via direct binding with TIAF1 (Pierrat et al., 2001) (**Figure 4G**). Aggregation of SH3GLB2 can be found in the brain cortex and hippocampus (Lee et al., 2017).

### WWOX Controls TRAPPC6AΔ, TIAF1, and Tau Aggregation *in vivo*: Effect of TGF-β

The WWOX physically binds TPC6AΔ, TIAF1, and Tau and prevents their aggregation (Lee et al., 2010; Chang and Chang, 2015; Chang et al., 2015; Sze et al., 2015). TGF-β induces the dissociation between WWOX and TIAF1, or TPC6AΔ. The dissociated TPC6AΔ/TIAF1 aggregates cause caspase activation, APP degradation, and ultimately formation of amyloid β (Lee et al., 2010; Chang and Chang, 2015; Chang et al., 2015). Similarly, when WWOX protein expression is downregulated, TPC6AΔ polymerizes first and then binds TIAF1 to induce further polymerization (Chang and Chang, 2015; Chang et al., 2015; Sze et al., 2015) (**Figure 4G**).

Also, knockdown of WWOX by small interfering RNA (siRNA) induces spontaneous aggregation of TPC6AΔ and TIAF1 *in vitro*. Knockdown of TPC6AΔ fails to cause TIAF1 aggregation (Chang and Chang, 2015), suggesting that TPC6AΔ aggregates first, followed by TIAF1 aggregation. Collectively, when WWOX is significantly downregulated, TPC6AΔ becomes phosphorylated at Ser35 and forms aggregates in the nucleus, followed by relocating to the mitochondria to bind TIAF1 and both proteins become aggregated (Chang and Chang, 2015; Sze et al., 2015) (**Figure 4G**). Thus, one line of *in vitro* evidence reveals that without WWOX, the TPC6AΔ/TIAF1 aggregates cause formation of extracellular amyloid β and intracellular Tau aggregates (Lee et al., 2010; Chang and Chang, 2015; Chang et al., 2015). Further, *in vivo* evidence revealed that when *Wwox* gene is knocked out in mice, aggregation of TIAF1, TPC6AΔ, amyloid β, Tau, and many other proteins occurs in the brains in less than 3 weeks (Chang and Chang, 2015; Chang et al., 2015) (**Figures 4A–E**). Taken together, WWOX plays a role in limiting protein aggregation *in vivo*.

### WWOX Phosphorylation at Ser14 and Its Potential Role in Neurodegeneration

Site-specific WWOX phosphorylation is associated with cell differentiation and many other events (Huang et al., 2016; Huang and Chang, 2018). During forced cell differentiation, WWOX rapidly undergoes phosphorylation at Ser14 in leukemia cells (Huang et al., 2016; Huang and Chang, 2018) and in diseased organs (Lee et al., 2017). pS14-WWOX does not cause apoptosis. In contrast, overly expressed pY33-WWOX induces apoptosis (Chang et al., 2007). It suggests that the levels of pS14-WWOX and pY33-WWOX must be in a good balance *in vivo*. Under stress conditions, WWOX is phosphorylated at Tyr33 to induce apoptosis. During cell differentiation or disease progression (e.g., AD), WWOX is phosphorylated at Ser14 (Huang and Chang, 2018).

Ten-month-old triple transgenic (3xTg) mice for AD develop memory loss probably due, in part, to accumulated aggregates of TPC6AΔ, SH3GLB2, tau and Aβ, along with inflammatory NF-κB activation, in the hippocampal and cortical areas (Lee et al., 2017). Notably, significantly increased phosphorylation of WWOX at Ser14, but not Tyr33, is shown in their brain lesions (Lee et al., 2017). Zfra blocks Ser14 phosphorylation in WWOX, significantly reduces accumulation of TPC6AΔ, SH3GLB2, tau and Aβ aggregates, suppresses NF-κB activation, and restores memory in these mice (Lee et al., 2017). *In vitro* analysis showed that Zfra binds cytosolic proteins for accelerating their degradation in ubiquitin/proteasome-independent manner (Lee et al., 2017).

B16F10 melanoma-growing nude mice develop neuronal death in the hippocampus, amyloid plaque formation in the cortex, and melanoma infiltration in the lung in less than 2 months (Lee et al., 2017). Zfra inhibits pS14-WWOX expression in the lung and brain lesions, clears up cortical plaques, and thereby suppresses cancer growth and neuronal death (Lee et al., 2017). Together, WWOX phosphorylation at Ser14 supports the progression of neurodegeneration in the hippocampus and plaque formation in the cortex, as well as cancer progression (Huang and Chang, 2018).

### Is WWOX a Molecular Chaperone?

WWOX retards neurodegeneration pathology by binding and blocking GSK-3β, ERK, JNK and probably other kinases and enhancing neurite outgrowth and neuronal differentiation (Sze et al., 2004; Wang et al., 2012). WWOX probably functions as a protein chaperone to prevent protein misfolding and degradation by the ubiquitin/proteasome system. Under stress conditions, activated WWOX with Tyr33 phosphorylation binds p53, and both proteins work synergistically to induce apoptosis (Chang et al., 2005a). Without binding, p53 relocates to the cytoplasm and undergoes degradation (Chang et al., 2005a). It has been proposed that the second WW domain of WWOX is an orphan module devoid of ligand binding function but is a chaperone necessary to stabilize the first WW domain in conducting protein/protein interactions (Farooq, 2015).

## Sex Steroid Hormones in Neuroprotection

Sex steroid hormones are decreased in menopause women and aged men. Deficiency of 17-β-estradiol (E2), a major form of estrogens, is implicated in age-related cognitive decline in human and non-human primates. Estrogens modulate hippocampal synaptic spine growth, structural plasticity, and neuronal excitability, which affect long-term potentiation in learning and memory (Teyler et al., 1980; Brinton, 1993; Warren et al., 1995; Engler-Chiurazzi et al., 2016; Muñoz-Mayorga et al., 2018).

Decreased serum sex steroid hormone levels in postmenopausal women or in aged men increase the risk for developing NDs. Participation of steroid sex hormones in neuroprotection through the interaction of E2 and estrogen receptors (ER) during brain injury and neurodegeneration has been extensively investigated and very well reviewed (Brann et al., 2007; Arevalo et al., 2015; Engler-Chiurazzi et al., 2016).

There are two classes of ERs, namely nuclear and membrane receptors. Upon stimulation with estrogens, ERα and ERβ translocate to the nucleus, bind chromosomal DNA, and function as transcription factors (Shang et al., 2000; Safe and Kim, 2008; Carroll, 2016). Membrane estrogen receptors (mERs) are mostly G protein-coupled receptors and are responsible for transducing signals upon stimulating with an estrogen. Known mERs are GPR30, ER-X, and G_q_-mER. During signaling, ERα and ERβ translocate to the nucleus and bind estrogen-responsive elements (EREs) in the promoter regions of specific genes to recruit transcriptional co-activators and co-repressors to control gene transcription (Shang et al., 2000; Safe and Kim, 2008; Carroll, 2016). Alternatively, ERs act as transcriptional partners at non-ERE sites. ERs are also associated with plasma membrane lipid rafts to bind neurotransmitters and proteins, which drives the growth factor receptor signaling to interact with other neuroprotective signaling pathways or elicit redundant neuroprotection signaling (e.g., PI3K-AKT, ERK1-ERK2, and JAK-STAT3) (Ramírez et al., 2009; Arevalo et al., 2015).

### ER Protective Signaling Pathways

**FIGURE 5:**
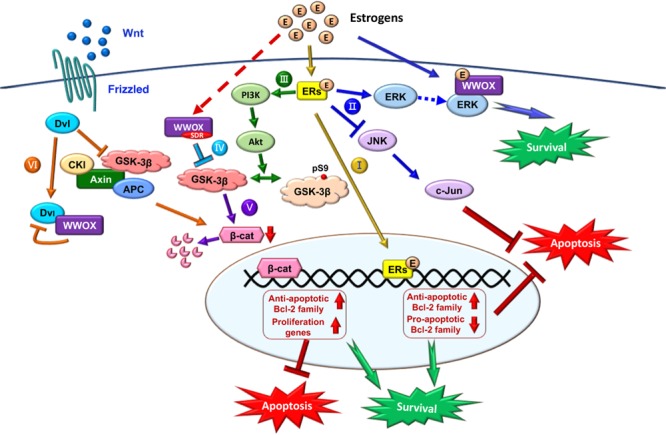
Role of E2/ER/WWOX in initiating protective pathways. The pathways include: Route I, E2/ER-mediated upregulation of antiapoptotic Bcl-2 family proteins, and downregulation of proapoptotic Bcl-2 family members (Yao et al., 2007) (see the route in yellow line). Route II, Activation of the pro-survival ERK/WWOX and PI3K/Akt signaling cascades to block the pro-apoptotic JNK signaling and protect the neural tissues from damages (Tang et al., 2014) (route in blue). Route III, E2 activates PI3K via ERα and mERs, followed by activating Akt to phosphorylate GSK-3β at Ser9 for functional inactivation (Ruiz-Palmero et al., 2013) (route in green). Route IV, The SDR domain of WWOX binds and limits GSK-3β activity for neuroprotection (Wang et al., 2012) (route in light blue). Route V, Suppression of GSK-3β (e.g., by WWOX) leads to a reduced β-catenin degradation, which is regulated by E2 through the ERα/PI3K/AKT/GSK-3β signaling pathway (Perez-Alvarez et al., 2012) (route in purple). Route VI, In the Wnt/Frizzled signaling pathway, Wnt protein induces the activation of Dvl to block the activity of GSK-3β. Without Wnt, β-catenin is subjected to destruction by the complex of axin, APC, CK1α, and GSK-3β (Bouteille et al., 2009). Transiently overexpressed WWOX binds Dvl to suppress the Wnt signaling (Bouteille et al., 2009) (route in orange).

Regarding the protective signaling pathways, ERs activate the ERK and PI3K signaling cascades, which leads to the inhibition of pro-apoptotic JNK signaling and thereby protects neural tissues from damages (Mannella and Brinton, 2006; Jover-Mengual et al., 2010; Tang et al., 2014). E2/ER signaling suppresses apoptosis by upregulating antiapoptotic Bcl-2 and family proteins, and downregulating proapoptotic Bcl-2 family members (**Figure 5**) (Koski et al., 2004; Yao et al., 2007). E2 activates PI3K via ERα and mERs. Phosphorylated PI3K activates Akt, followed by Akt phosphorylating GSK-3β at Ser9 to decrease GSK-3β activity (**Figure 5**) (Cardona-Gomez et al., 2004; Ruiz-Palmero et al., 2013). The inhibition of GSK-3β activity is a common mechanism of neuroprotection by several factors including WWOX (Cardona-Gomez et al., 2004; Sze et al., 2004; de Paula et al., 2009; Perez-Alvarez et al., 2012; Wang et al., 2012). GSK-3β inhibition-induced neuroprotection also involves β-catenin, which is regulated by E2 through the ERα/PI3K/AKT/GSK3β signaling pathway (**Figure 5**) (de Paula et al., 2009; Perez-Alvarez et al., 2012). Together, these observations suggest that E2/ERs-mediated neuroprotection is mediated through the interactions of ERs with the pathways induced by different neuroprotective signaling pathways or the protective effects of growth factors.

## WWOX as a Receptor for Sex Steroid Hormones for Signaling

The WWOX is a potential cytosolic or membrane receptor for sex steroid hormones (Chang et al., 2005b; Su et al., 2012). WWOX is highly expressed in hormone- or enzyme-secreting organs. WWOX is most abundant in the ductal epithelial cells such as in the breast and prostate. WWOX controls the growth and progression of breast and prostate cancers (Bednarek et al., 2000; Chang et al., 2005b; Nunez et al., 2005; O’Keefe et al., 2011). Loss of WWOX accelerates cancer growth and metastasis. The SDR domain of WWOX is associated with aerobic metabolism and control of the generation of reactive oxygen species (O’Keefe et al., 2011; Choo et al., 2015), which is crucial in limiting the progression of neurodegeneration (Su et al., 2012; Chang et al., 2014).

### Estrogens and Androgens Bind the SDR Domain of WWOX

Estrogens or androgens bind the NSYK (Asn-Ser-Tyr-Lys) motif in the *C*-terminal SDR domain of WWOX (Chang et al., 2005b; Su et al., 2012). This binding causes nuclear accumulation of activated or Tyr33-phosphorylated WWOX (pY33-WWOX) (Chang et al., 2005b; Su et al., 2012). Excessive accumulation of pY33-WWOX in the nucleus induces apoptosis. Notably, estrogen or androgen-mediated WWOX activation is independent of ERs or androgen receptor (AR), suggesting that WWOX by itself acts as a receptor (Chang et al., 2005b; Su et al., 2012). WWOX expression is significantly upregulated during the early stage of normal prostate and breast tissue progression toward hyperplasia and cancerous stages (Chang et al., 2005b). Upon reaching metastatic stage, cancer cells do not express WWOX due, in part, to hypermethylation at the promoter region.

Indeed, the expression levels of WWOX positively correlate with the hormone receptor status, but negatively correlate with the clinical stages of breast and ovarian cancers (Chang et al., 2005b; Guler et al., 2011). Loss of WWOX confers resistance to tamoxifen due to upregulation of ER and human epidermal growth factor receptor 2 (Her2) and their transcriptional activities (Guler et al., 2007; Salah et al., 2010). Tamoxifen is one of the estrogen receptor modulators, which regulates hormone-secreting tissue activities for treatment and prevention of ER-positive cancers. Together, these observations suggest WWOX functions as an enzyme or a receptor involved in sex steroid metabolism to modulate disease progression.

### Crosstalk of ERs, WWOX, and Wnt Signaling

Shown in the **Table 1** is a comparison between WWOX and ERs/mERs regarding their molecular structures, actions and potential mechanisms. WWOX is involved in many signal pathways (Chang et al., 2007, 2010, 2014; Chang, 2015; Huang and Chang, 2018), and this allows its crosstalk with the signaling from ERs and mERs. For example, ERs activate the ERK1/2 and PI3K signaling cascades (Mannella and Brinton, 2006; Jover-Mengual et al., 2010; Tang et al., 2014), and that WWOX physically binds ERK1/2 for supporting cell survival (Lin et al., 2011) (**Figure 5**) and lymphocyte differentiation (Huang et al., 2016).

**Table 1:** Functional comparison between ER and WWOX in neurodegeneration.

		Estrogen receptor	WWOX
Gene		ERα-6q25.1 (Hattori et al., 2016); ERβ-14q23.2 (Al-Nakhle et al., 2013)	16q23 (Bednarek et al., 2000; Ried et al., 2000)
Localization in cell		Plasma membrane, cytosol, mitochondria, nucleus. (Shang et al., 2000; Safe and Kim, 2008; Tang et al., 2014)	Plasma membrane, cytosol, mitochondria, nucleus. (Chang et al., 2007; Hsu et al., 2009, 2016)
Role in embryonic development		ER impact on the development of reproductive system, liver, muscle, pancreas, blood, and brain. (Bondesson et al., 2015)	Loss of WWOX related to defects in bone formation and steroidogenesis, and abnormal neural and sexual development. (Ludes-Meyers et al., 2009; Abu-Remaileh and Aqeilan, 2015)
Role in neural system	Alzheimer’s disease	Inhibition of Tau hyperphosphorylation by activating PI3K/Akt to reduce GSK3β activity. (Cardona-Gomez et al., 2004; Ramírez et al., 2009; Arevalo et al., 2015)	Down-regulation of Tau phosphorylation through interacting with GSK-3β. (Sze et al., 2004; Chang et al., 2007; Wang et al., 2012)
	Injury	As a pro-survival factor by up-regulating Wnt/β-catenin signaling, PI3 kinase/Akt signaling, anti-apoptotic Bcl-2, and inhibiting microglia activation. (Dubal et al., 1999; Quesada et al., 2008; Zhang et al., 2008; Perez-Alvarez et al., 2012)	As a pro-apoptotic factor through interacting with CREB, JNK, NF-κB, p53. (Chang et al., 2007; Li et al., 2009)
Metabolic syndromes		Aberrant ERs related to imbalance of energy metabolism, homeostasis of lipid and glucose, distribution of fat, and type II diabetes. (Brann et al., 2007; Hevener et al., 2015; Zeng et al., 2016)	Deficiency in WWOX associated with hypertension, hypoglycemia, hypocalcemia, metabolic acidosis, type II diabetes, and homeostasis of lipid and steroids. (Chang et al., 2005b, 2007; Nunez et al., 2005; Ludes-Meyers et al., 2009; O’Keefe et al., 2011; Chang et al., 2014)

During the signaling, E2/ER upregulates the antiapoptotic Bcl-2 family proteins and downregulates the proapoptotic Bcl-2 family members (Yao et al., 2007) (Route I, **Figure 5**). Also, E2/ER-mediated activation of the ERK and PI3K/Akt signaling cascades suppresses the JNK signaling for neuroprotection (Tang et al., 2014) (Route II, **Figure 5**).

Also, E2/ER activates PI3K and Akt to inactivate GSK-3β by phosphorylating GSK-3β at Ser9 (Ruiz-Palmero et al., 2013) (Route III, **Figure 5**). WWOX via its SDR domain binds and limits GSK-3β activity (Wang et al., 2012) (Route IV, **Figure 5**), and this leads to a reduced β-catenin degradation regulated by E2 via the ERα/PI3K/AKT/GSK-3β signaling pathway (Perez-Alvarez et al., 2012) (Route V, **Figure 5**). GSK-3β initiates the proteasomal degradation of β-catenin by phosphorylating β-catenin on key residues (**Figure 5**). In addition, nuclear GSK-3β binds β-catenin, without causing β-catenin phosphorylation and degradation, but reduces the activity of β-catenin/TCF-dependent transcription via GSK-3β-Axin binding (Caspi et al., 2008). GSK-3β inhibits competitive phosphorylation of β-catenin and hence facilitates the function of β-catenin, thus enabling cells with phosphorylated Tau to escape apoptosis (Li et al., 2007).

The Wnt signaling pathway modulates amyloid β peptide-mediated neuropathology in AD by inactivating GSK-3β, which in turn prevents Tau phosphorylation (Bhat and Budd, 2002; Boonen et al., 2009) (Route VI, **Figure 5**). Wnt protein induces the activation of Dvl in the Wnt/β-catenin signaling to block the activity of GSK-3β. In the absence of Wnt ligand, β-catenin is destructed by the complex of axin, APC, CK1α, and GSK-3β (Bhat and Budd, 2002; Boonen et al., 2009). Ectopic WWOX sequesters cytosolic disheveled family protein (Dvl) and thus inhibits the Wnt/β-catenin pathway (Bouteille et al., 2009; Huang and Chang, 2018). WWOX suppresses the c-Jun transcriptional activity and cAMP response element-binding protein (CREB) function, which is associated with amyloid depositions (Savage et al., 2002; Li et al., 2009).

### Estrogen in the pY33-WWOX/pS15-p53 Complex

Catechol estrogens have been shown to covalently conjugate with serum proteins from diabetic patients – the so-called “estrogenization” (Ku et al., 2016). When insulin is estrogenized, its receptor-binding pocket is blocked, thus resulting in functional blockade (Ku et al., 2016). The conserved NSYK motif in the SDR domain of WWOX is capable of interacting with androgens and estrogens and other proteins (Chang et al., 2007; Su et al., 2012). At micromolar levels, exogenous E2 binds WWOX and induces activation of both WWOX and p53 via phosphorylation at Tyr33 and Ser15, respectively, in COS7 fibroblasts (Chang et al., 2007; Su et al., 2012). Excessive accumulation of the E2/pY33-WWOX/pS15-p53 complex in the nucleus results in cell death (Chang et al., 2007; Su et al., 2012). JNK1 blocks the apoptotic function of overly expressed WWOX (Chang et al., 2003a).

## Perspectives

### A Focus on WWOX and Protein Aggregation in Middle Age

Both aggregated tau and Aβ are considered as the key pathological markers of AD, and have been the center of focus for drug development over the past several decades. Aggregated tau and Aβ are usually found in the brain of AD patients over 70 years old while normal individuals from 40–70 years old possess very low amounts of aggregated tau and Aβ. We have determined the presence of aggregated proteins such as TPC6AD and TIAF1 in approximately 50% of the brains of mid-aged normal humans (Chang and Chang, 2015; Chang et al., 2015; Sze et al., 2015). Indeed, WWOX downregulation causes self-aggregation of TPC6AD and TIAF1 *in vitro* (Lee et al., 2010; Chang and Chang, 2015; Chang et al., 2015). *Wwox* gene knockout mice rapidly exhibit aggregation of many proteins in the brains just in 15 days after birth. These proteins include TPC6AD, TIAF1, and SH3GLB2, tau and Aβ (Lee et al., 2017). Notably, human newborns with *WWOX* deficiency rapidly develop severe neural diseases, metabolic disorders, retarded growth and early death. While TRAPPC6AΔ and TIAF1 are starters for protein aggregation, these proteins are indeed potential targets for drug development. Development of therapeutic peptides and humanized monoclonal antibodies is under way.

### Zfra Initiates a Novel Immune Response to Block Protein Aggregation and Restores Memory Loss

Zfra restores memory deficits in Alzheimer’s disease triple-transgenic mice by blocking the aggregation of TPC6AΔ, SH3GLB2, Tau, and amyloid β, and reducing inflammatory NF-κB activation (Lee et al., 2017). As a WWOX-binding protein, exogenous Zfra peptide, when introduced in the circulation in mice, is mainly deposited in the spleen. Zfra binds membrane hyaluronidase Hyal-2 in non-T/non-B Z lymphocytes. Z cells then become activated to suppress cancer growth (Lee et al., 2015). Intriguingly, Z cells exhibit a memory function in killing cancer cells, even though these cells have never exposed to the cancer cells. Autologous Z cells, once activated by Zfra, are of great therapeutic use in treating cancer and probably neurodegeneration such as AD. Both full-length Zfra and a truncated 7-amino-acid Zfra4-10 are effective in suppressing cancer growth (Lee et al., 2015) and restoring memory loss (Lee et al., 2017). Since Zfra is stably retained on the Z cell surface, Zfra activates the Hyal-2/WWOX/Smad4 signaling in Z cells. Peptides or monoclonal antibodies are being developed to target membrane Hyal-2 as well as WWOX and to activate Z cells in blocking cancer and neurodegeneration.

### A pTyr33-WWOX Peptide as an Agent for Blocking Neuronal Injury and Death

Finally, an 11-amino-acid phospho-Try33 WWOX peptide was developed to block neurotoxin MPP+-induced neuronal death in the brain (Lo et al., 2008). This phospho-peptide effectively suppresses neuronal death via inhibition of JNK1 activation. In controls, non-phospho-WWOX peptide has no effect. The phospho-Try33 WWOX peptide is now being tested for its efficacy in blocking neuronal death in AD and traumatic brain injury.

## Author Contributions

C-CL and C-CT carried out the literature review. Y-AC, C-CL, P-CH, and N-SC prepared schematic graphs. C-HC reviewed and revised the manuscript. C-IS and N-SC wrote the manuscript. N-SC completed the final version and provided rebuttal letters to all reviewers. All authors read and approved the final manuscript.

## Conflict of Interest Statement

The authors declare that the research was conducted in the absence of any commercial or financial relationships that could be construed as a potential conflict of interest. The reviewer K-JT declared a shared affiliation, with no collaboration, with several of the authors, C-CL, C-CT, Y-AC, P-CH, C-HC, S-NW, N-SC, C-IS, I-TL, to the handling Editor at the time of the review.

## Footnotes

1 http://www.brain-map.org

2 https://www.genecards.org/cgi-bin/carddisp.pl?gene=WWOX

## Supplementary Material

TABLE S1 Functional comparison between ER and WWOX in neurodegeneration.

## References

[B1] Abu-Remaileh M. Aqeilan R. I. (2015). The tumor suppressor WW domain-containing oxidoreductase modulates cell metabolism. *Exp. Biol. Med.* 240 345–350. 10.1177/1535370214561956 25491415 PMC4935230

[B2] Abu-Remaileh M. Joy-Dodson E. Schueler-Furman O. Aqeilan R. I. (2015). Pleiotropic functions of tumor suppressor WWOX in normal and cancer cells. *J. Biol. Chem.* 290 30728–30735. 10.1074/jbc.R115.676346 26499798 PMC4692203

[B3] Aderca I. Moser C. D. Veerasamy M. Bani-Hani A. H. Bonilla-Guerrero R. Ahmed K. (2008). The JNK inhibitor SP600129 enhances apoptosis of HCC cells induced by the tumor suppressor WWOX. *J. Hepatol.* 49 373–383. 10.1016/j.jhep.2008.05.015 18620777 PMC2574998

[B4] Aldaz C. M. Ferguson B. W. Abba M. C. (2014). WWOX at the crossroads of cancer, metabolic syndrome related traits and CNS pathologies. *Biochim. Biophys. Acta* 1846 188–200. 10.1016/j.bbcan.2014.06.001 24932569 PMC4151823

[B5] Alkhateeb A. M. Aburahma S. K. Habbab W. Thompson I. R. (2016). Novel mutations in *WWOX, RARS2*, and *C10orf2* genes in consanguineous Arab families with intellectual disability. *Metab. Brain Dis.* 31 901–907. 10.1007/s11011-016-9827-9 27121845

[B6] Al-Nakhle H. Smith L. Bell S. M. Burns P. A. Cummings M. Hanby A. M. (2013). Regulation of estrogen receptor β1 expression in breast cancer by epigenetic modification of the 5′ regulatory region. *Int. J. Oncol.* 43 2039–2045. 10.3892/ijo.2013.2112 24068253

[B7] Aqeilan R. I. Pekarsky Y. Herrero J. J. Palamarchuk A. Letofsky J. Druck T. (2004). Functional association between Wwox tumor suppressor protein and p73, a p53 homolog. *Proc. Natl. Acad. Sci. U.S.A.* 101 4401–4406. 10.1073/pnas.0400805101 15070730 PMC384759

[B8] Arevalo M. A. Azcoitia I. Garcia-Segura L. M. (2015). The neuroprotective actions of oestradiol and oestrogen receptors. *Nat. Rev. Neurosci.* 16 17–29. 10.1038/nrn3856 25423896

[B9] Augustinack J. C. Schneider A. Mandelkow E. M. Hyman B. T. (2002). Specific tau phosphorylation sites correlate with severity of neuronal cytopathology in Alzheimer’s disease. *Acta Neuropathol.* 103 26–35. 10.1007/s004010100423 11837744

[B10] Avila J. Wandosell F. Hernandez F. (2010). Role of glycogen synthase kinase-3 in Alzheimer’s disease pathogenesis and glycogen synthase kinase-3 inhibitors. *Expert. Rev. Neurother.* 10 703–710. 10.1586/ern.10.40 20420491

[B11] Bains M. Hall E. D. (2012). Antioxidant therapies in traumatic brain and spinal cord injury. *Biochim. Biophys. Acta* 1822 675–684. 10.1016/j.bbadis.2011.10.017 22080976 PMC4134010

[B12] Bednarek A. K. Laflin K. J. Daniel R. L. Liao Q. Hawkins K. A. Aldaz C. M. (2000). WWOX, a novel WW domain-containing protein mapping to human chromosome 16q23.3-24.1, a region frequently affected in breast cancer. *Cancer Res.* 60 2140–2145. 10786676

[B13] Bhat R. V. Budd S. L. (2002). GSK3β signalling: casting a wide net in Alzheimer’s disease. *Neurosignals* 11 251–261. 10.1159/000067423 12566926

[B14] Bondesson M. Hao R. Lin C. Y. Williams C. Gustafsson J. A. (2015). Estrogen receptor signaling during vertebrate development. *Biochim. Biophys. Acta* 1849 142–151. 10.1016/j.bbagrm.2014.06.005 24954179 PMC4269570

[B15] Boonen R. A. van Tijn P. Zivkovic D. (2009). Wnt signaling in Alzheimer’s disease: up or down, that is the question. *Ageing Res. Rev.* 8 71–82. 10.1016/j.arr.2008.11.003 19101658

[B16] Bouteille N. Driouch K. Hage P. E. Sin S. Formstecher E. Camonis J. (2009). Inhibition of the Wnt/beta-catenin pathway by the WWOX tumor suppressor protein. *Oncogene* 28 2569–2580. 10.1038/onc.2009.120 19465938

[B17] Brann D. W. Dhandapani K. Wakade C. Mahesh V. B. Khan M. M. (2007). Neurotrophic and neuroprotective actions of estrogen: basic mechanisms and clinical implications. *Steroids* 72 381–405. 10.1016/j.steroids.2007.02.003 17379265 PMC2048656

[B18] Brinton R. D. (1993). 17beta-Estradiol induction of filopodial growth in cultured hippocampal neurons within minutes of exposure. *Mol. Cell. Neurosci.* 4 36–46. 10.1006/mcne.1993.1005 19912906

[B19] Cardona-Gomez P. Perez M. Avila J. Garcia-Segura L. M. Wandosell F. (2004). Estradiol inhibits GSK3 and regulates interaction of estrogen receptors, GSK3, and beta-catenin in the hippocampus. *Mol. Cell. Neurosci.* 25 363–373. 10.1016/j.mcn.2003.10.008 15033165

[B20] Carroll J. S. (2016). Mechanisms of oestrogen receptor (ER) gene regulation in breast cancer. *Eur. J. Endocrinol.* 175 R41–R49. 10.1530/EJE-16-r0124 26884552 PMC5065078

[B21] Caspi M. Zilberberg A. Eldar-Finkelman H. Rosin-Arbesfeld R. (2008). Nuclear GSK-3β inhibits the canonical Wnt signalling pathway in a beta-catenin phosphorylation-independent manner. *Oncogene* 27 3546–3555. 10.1038/sj.onc.1211026 18223684

[B22] Chang H. T. Liu C. C. Chen S. T. Yap Y. V. Chang N. S. Sze C. I. (2014). WW domain-containing oxidoreductase in neuronal injury and neurological diseases. *Oncotarget* 5 11792–11799. 10.18632/oncotarget.2961 25537520 PMC4322972

[B23] Chang J. Y. Chang N. S. (2015). WWOX dysfunction induces sequential aggregation of TRAPPC6ADelta, TIAF1, tau and amyloid beta, and causes apoptosis. *Cell Death Discov.* 1:15003. 10.1038/cddiscovery.2015.3 27551439 PMC4981022

[B24] Chang J. Y. Chiang M. F. Lin S. R. Lee M. H. He H. Chou P. Y. (2012). TIAF1 self-aggregation in peritumor capsule formation, spontaneous activation of SMAD-responsive promoter in p53-deficient environment, and cell death. *Cell Death Dis.* 3:e302. 10.1038/cddis.2012.36 22534828 PMC3358014

[B25] Chang J. Y. He R. Y. Lin H. P. Hsu L. J. Lai F. J. Hong Q. (2010). Signaling from membrane receptors to tumor suppressor WW domain-containing oxidoreductase. *Exp. Biol. Med.* 235 796–804. 10.1258/ebm.2010.009351 20542955

[B26] Chang J. Y. Lee M. H. Lin S. R. Yang L. Y. Sun H. S. Sze C. I. (2015). Trafficking protein particle complex 6A delta (TRAPPC6ADelta) is an extracellular plaque-forming protein in the brain. *Oncotarget* 6 3578–3589. 10.18632/oncotarget.2876 25650666 PMC4414138

[B27] Chang N. S. (2002). A potential role of p53 and WOX1 in mitochondrial apoptosis (review). *Int. J. Mol. Med.* 9 19–24. 10.3892/ijmm.9.1.19 11744990

[B28] Chang N. S. (2015). Introduction to a thematic issue for WWOX. *Exp. Biol. Med.* 240 281–284. 10.1177/1535370215574226 25802472 PMC4935221

[B29] Chang N. S. (2016). Bubbling cell death: a hot air balloon released from the nucleus in the cold. *Exp. Biol. Med.* 241 1306–1315. 10.1177/1535370216644531 27075929 PMC4950269

[B30] Chang N. S. Doherty J. Ensign A. (2003a). JNK1 physically interacts with WW domain-containing oxidoreductase (WOX1) and inhibits WOX1-mediated apoptosis. *J. Biol. Chem.* 278 9195–9202. 10.1074/jbc.M208373200 12514174

[B31] Chang N. S. Doherty J. Ensign A. Lewis J. Heath J. Schultz L. (2003b). Molecular mechanisms underlying WOX1 activation during apoptotic and stress responses. *Biochem. Pharmacol.* 66 1347–1354. 14555208 10.1016/s0006-2952(03)00484-2

[B32] Chang N. S. Doherty J. Ensign A. Schultz L. Hsu L. J. Hong Q. (2005a). WOX1 is essential for tumor necrosis factor-, UV light-, staurosporine-, and p53-mediated cell death, and its tyrosine 33-phosphorylated form binds and stabilizes serine 46-phosphorylated p53. *J. Biol. Chem.* 280 43100–43108. 10.1074/jbc.M505590200 16219768

[B33] Chang N. S. Schultz L. Hsu L. J. Lewis J. Su M. Sze C. I. (2005b). 17beta-Estradiol upregulates and activates WOX1/WWOXv1 and WOX2/WWOXv2 in vitro: potential role in cancerous progression of breast and prostate to a premetastatic state in vivo. *Oncogene* 24 714–723. 10.1038/sj.onc.1208124 15580310

[B34] Chang N. S. Hsu L. J. Lin Y. S. Lai F. J. Sheu H. M. (2007). WW domain-containing oxidoreductase: a candidate tumor suppressor. *Trends Mol. Med.* 13 12–22. 10.1016/j.molmed.2006.11.006 17142102

[B35] Chang N. S. Pratt N. Heath J. Schultz L. Sleve D. Carey G. B. (2001). Hyaluronidase induction of a WW domain-containing oxidoreductase that enhances tumor necrosis factor cytotoxicity. *J. Biol. Chem.* 276 3361–3370. 10.1074/jbc.M007140200 11058590

[B36] Chen S. J. Lin P. W. Lin H. P. Huang S. S. Lai F. J. Sheu H. M. (2015). UV irradiation/cold shock-mediated apoptosis is switched to bubbling cell death at low temperatures. *Oncotarget* 6 8007–8018. 10.18632/oncotarget.3153 25779665 PMC4480731

[B37] Chen S. T. Chuang J. I. Cheng C. L. Hsu L. J. Chang N. S. (2005). Light-induced retinal damage involves tyrosine 33 phosphorylation, mitochondrial and nuclear translocation of WW domain-containing oxidoreductase in vivo. *Neuroscience* 130 397–407. 10.1016/j.neuroscience.2004.07.054 15664696

[B38] Chen S. T. Chuang J. I. Wang J. P. Tsai M. S. Li H. Chang N. S. (2004). Expression of WW domain-containing oxidoreductase WOX1 in the developing murine nervous system. *Neuroscience* 124 831–839. 10.1016/j.neuroscience.2003.12.036 15026124

[B39] Chételat G. (2018). Multimodal neuroimaging in Alzheimer’s disease: early diagnosis, physiopathological mechanisms, and impact of lifestyle. *J. Alzheimers Dis.* 64 S199–S211. 10.3233/JAD-179920 29504542 PMC6004909

[B40] Choo A. O’Keefe L. V. Lee C. S. Gregory S. L. Shaukat Z. Colella A. (2015). Tumor suppressor WWOX moderates the mitochondrial respiratory complex. *Genes Chromosomes Cancer* 54 745–761. 10.1002/gcc.22286 26390919

[B41] Dayan S. O’Keefe L. V. Choo A. Richards R. I. (2013). Common chromosomal fragile site FRA16D tumor suppressor WWOX gene expression and metabolic reprograming in cells. *Genes Chromosomes Cancer* 52 823–831. 10.1002/gcc.22078 23765596

[B42] de Paula V. J. R. Guimaraes F. M. Diniz B. S. Forlenza O. V. (2009). Neurobiological pathways to Alzheimer’s disease: amyloid-β, TAU protein or both? *Dement. Neuropsychol.* 3 188–194. 10.1590/S1980-57642009DN30300003 29213627 PMC5618972

[B43] Dubal D. B. Shughrue P. J. Wilson M. E. Merchenthaler I. Wise P. M. (1999). Estradiol modulates bcl-2 in cerebral ischemia: a potential role for estrogen receptors. *J. Neurosci.* 19 6385–6393. 10.1523/JNEUROSCI.19-15-06385 10414967 PMC6782804

[B44] Dugger B. N. Dickson D. W. (2016). Pathology of Neurodegenerative Diseases. *Cold Spring Harb. Perspect. Biol.* 9:a028035. 10.1101/cshperspect.a028035 28062563 PMC5495060

[B45] Elsaadany L. El-Said M. Ali R. Kamel H. Ben-Omran T. (2016). W44X mutation in the WWOX gene causes intractable seizures and developmental delay: a case report. *BMC Med. Genet.* 17:53. 10.1186/s12881-016-0317-z 27495153 PMC4975905

[B46] Engler-Chiurazzi E. B. Singh M. Simpkins J. W. (2016). Reprint of: from the 90s to now: a brief historical perspective on more than two decades of estrogen neuroprotection. *Brain Res.* 1645 79–82. 10.1016/j.brainres.2016.06.016 27317847 PMC4969093

[B47] Farooq A. (2015). Structural insights into the functional versatility of WW domain-containing oxidoreductase tumor suppressor. *Exp. Biol. Med.* 240 361–374. 10.1177/1535370214561586 25662954 PMC4374002

[B48] Gaudio E. Palamarchuk A. Palumbo T. Trapasso F. Pekarsky Y. Croce C. M. (2006). Physical association with WWOX suppresses c-Jun transcriptional activity. *Cancer Res.* 66 11585–11589. 10.1158/0008-5472.CAN-06-3376 17178850

[B49] Guler G. Himmetoglu C. Jimenez R. E. Geyer S. M. Wang W. P. Costinean S. (2011). Aberrant expression of DNA damage response proteins is associated with breast cancer subtype and clinical features. *Breast Cancer Res. Treat.* 129 421–432. 10.1007/s10549-010-1248-6 21069451 PMC3677189

[B50] Guler G. Iliopoulos D. Guler N. Himmetoglu C. Hayran M. Huebner K. (2007). Wwox and Ap2gamma expression levels predict tamoxifen response. *Clin. Cancer Res.* 13 6115–6121. 10.1158/1078-0432.CCR-07-1282 17947476

[B51] Gwynn B. Smith R. S. Rowe L. B. Taylor B. A. Peters L. L. (2006). A mouse TRAPP-related protein is involved in pigmentation. *Genomics* 88 196–203. 10.1016/j.ygeno.2006.04.002 16697553

[B52] Hamilton G. Harris S. E. Davies G. Liewald D. C. Tenesa A. Starr J. M. (2011). Alzheimer’s disease genes are associated with measures of cognitive ageing in the lothian birth cohorts of 1921 and 1936. *Int. J. Alzheimers Dis.* 2011:505984. 10.4061/2011/505984 21766012 PMC3132531

[B53] Hartl F. U. (2017). Protein misfolding diseases. *Annu. Rev. Biochem.* 86 21–26. 10.1146/annurev-biochem-061516-044518 28441058

[B54] Hartmann A. Muth C. Dabrowski O. Krasemann S. Glatzel M. (2017). Exosomes and the prion protein: more than one truth. *Front. Neurosci.* 11:194. 10.3389/fnins.2017.00194 28469550 PMC5395619

[B55] Hattori Y. Ishii H. Munetomo A. Watanabe H. Morita A. Sakuma Y. (2016). Human C-terminally truncated ERα variants resulting from the use of alternative exons in the ligand-binding domain. *Mol. Cell. Endocrinol.* 425 111–122. 10.1016/j.mce.2016.01.026 26835991

[B56] Henriques A. G. Vieira S. I. da Cruz e Silva E. F. da Cruz e Silva O. A. (2009). Alphabeta hinders nuclear targeting of AICD and Fe65 in primary neuronal cultures. *J. Mol. Neurosci.* 39 248–255. 10.1007/s12031-009-9192-9 19340611 PMC2744832

[B57] Hevener A. L. Clegg D. J. Mauvais-Jarvis F. (2015). Impaired estrogen receptor action in the pathogenesis of the metabolic syndrome. *Mol. Cell. Endocrinol.* 418(Pt 3) 306–321. 10.1016/j.mce.2015.05.020 26033249 PMC5965692

[B58] Higuchi-Sanabria R. Frankino P. A. Paul J. W. III Tronnes S. U. Dillin A. (2018). A futile battle? protein quality control and the stress of aging. *Dev. Cell* 44 139–163. 10.1016/j.devcel.2017.12.020 29401418 PMC5896312

[B59] Hong Q. Hsu L. J. Chou P. Y. Chou Y. T. Lu C. Y. Chen Y. A. (2013). Self-aggregating TIAF1 in lung cancer progression. *Transl. Respir. Med.* 1:5. 10.1186/2213-0802-1-5 27234387 PMC6733429

[B60] Hong Q. Hsu L. J. Schultz L. Pratt N. Mattison J. Chang N. S. (2007). Zfra affects TNF-mediated cell death by interacting with death domain protein TRADD and negatively regulates the activation of NF-kappaB, JNK1, p53 and WOX1 during stress response. *BMC Mol. Biol.* 8:50. 10.1186/1471-2199-8-50 17567906 PMC1904229

[B61] Hong Q. Sze C. I. Lin S. R. Lee M. H. He R. Y. Schultz L. (2009). Complement C1q activates tumor suppressor WWOX to induce apoptosis in prostate cancer cells. *PLoS One* 6:e5755. 10.1371/journal.pone.0005755 19484134 PMC2685983

[B62] Hsu L. J. Chiang M. F. Sze C. I. Su W. P. Yap Y. V. Lee I. T. (2016). HYAL-2-WWOX-SMAD4 signaling in cell death and anticancer response. *Front. Cell Dev. Biol.* 4:141. 10.3389/fcell.2016.00141 27999774 PMC5138198

[B63] Hsu L. J. Hong Q. Chen S. T. Kuo H. L. Schultz L. Heath J. (2017). Hyaluronan activates Hyal-2/WWOX/Smad4 signaling and causes bubbling cell death when the signaling complex is overexpressed. *Oncotarget* 8 19137–19155. 10.18632/oncotarget.13268 27845895 PMC5386674

[B64] Hsu L. J. Schultz L. Hong Q. Van Moer K. Heath J. Li M. Y. (2009). Transforming growth factor beta1 signaling via interaction with cell surface Hyal-2 and recruitment of WWOX/WOX1. *J. Biol. Chem.* 284 16049–16059. 10.1074/jbc.M806688200 19366691 PMC2708898

[B65] Huang S. S. Chang N. S. (2018). Phosphorylation/de-phosphorylation in specific sites of tumor suppressor WWOX and control of distinct biological events. *Exp. Biol. Med.* 243 137–147. 10.1177/1535370217752350 29310447 PMC5788152

[B66] Huang S. S. Su W. P. Lin H. P. Kuo H. L. Wei H. L. Chang N. S. (2016). Role of WW domain-containing oxidoreductase WWOX in driving T cell acute lymphoblastic leukemia maturation. *J. Biol. Chem.* 291 17319–17331. 10.1074/jbc.M116.716167 27339895 PMC5016130

[B67] Iatan I. Choi H. Y. Ruel I. Reddy M. V. Kil H. Lee J. (2014). The WWOX gene modulates high-density lipoprotein and lipid metabolism. *Circ. Cardiovasc. Genet.* 7 491–504. 10.1161/CIRCGENETICS.113.000248 24871327 PMC4315188

[B68] Jin C. Ge L. Ding X. Chen Y. Zhu H. Ward T. (2006). PKA-mediated protein phosphorylation regulates ezrin-WWOX interaction. *Biochem. Biophys. Res. Commun.* 341 784–791. 10.1016/j.bbrc.2006.01.023 16438931

[B69] Jover-Mengual T. Miyawaki T. Latuszek A. Alborch E. Zukin R. S. Etgen A. M. (2010). Acute estradiol protects CA1 neurons from ischemia-induced apoptotic cell death via the PI3K/Akt pathway. *Brain Res.* 1321 1–12. 10.1016/j.brainres.2010.01.046 20114038 PMC2836484

[B70] Koski C. L. Hila S. Hoffman G. E. (2004). Regulation of cytokine-induced neuron death by ovarian hormones: involvement of antiapoptotic protein expression and c-JUN N-terminal kinase-mediated proapoptotic signaling. *Endocrinology* 145 95–103. 10.1210/en.2003-0803 14512437

[B71] Ku M. C. Fang C. M. Cheng J. T. Liang H. C. Wang T. F. Wu C. H. (2016). Site-specific covalent modifications of human insulin by catechol estrogens: reactivity and induced structural and functional changes. *Sci. Rep.* 6:28804. 10.1038/srep28804 27353345 PMC4926285

[B72] Lai F. J. Cheng C. L. Chen S. T. Wu C. H. Hsu L. J. Lee J. Y. (2005). WOX1 is essential for UVB irradiation-induced apoptosis and down-regulated via translational blockade in UVB-induced cutaneous squamous cell carcinoma in vivo. *Clin. Cancer Res.* 11 5769–5777. 10.1038/cdd.2011.188 16115915

[B73] Lee J. C. Weissglas-Volkov D. Kyttala M. Dastani Z. Cantor R. M. Sobel E. M. (2008). WW-domain-containing oxidoreductase is associated with low plasma HDL-C levels. *Am. J. Hum. Genet.* 83 180–192. 10.1016/j.ajhg.2008.07.002 18674750 PMC2495060

[B74] Lee M. H. Lin S. R. Chang J. Y. Schultz L. Heath J. Hsu L. J. (2010). TGF-beta induces TIAF1 self-aggregation via type II receptor-independent signaling that leads to generation of amyloid β plaques in Alzheimer’s disease. *Cell Death Dis.* 1:e110. 10.1038/cddis.2010.83 21368882 PMC3032296

[B75] Lee M. H. Shih Y. H. Lin S. R. Chang J. Y. Lin Y. H. Sze C. I. (2017). Zfra restores memory deficits in Alzheimer’s disease triple-transgenic mice by blocking aggregation of TRAPPC6AΔ, SH3GLB2, tau, and amyloid β, and inflammatory NF-κB activation. *Alzheimers Dement* 3 189–204. 10.1016/j.trci.2017.02.001 29067327 PMC5651433

[B76] Lee M.-H. Su W.-P. Wang W.-J. Lin S.-R. Lu C.-Y. Chen Y.-A. (2015). Zfra activates memory Hyal-2+ CD3- CD19- spleen cells to block cancer growth, stemness, and metastasis *in vivo*. *Oncotarget* 6 3737–3751. 10.18632/oncotarget.2895 25686832 PMC4414150

[B77] Li H. L. Wang H. H. Liu S. J. Deng Y. Q. Zhang Y. J. Tian Q. (2007). Phosphorylation of tau antagonizes apoptosis by stabilizing beta-catenin, a mechanism involved in Alzheimer’s neurodegeneration. *Proc. Natl. Acad. Sci. U.S.A.* 104 3591–3596. 10.1073/pnas.0609303104 17360687 PMC1805527

[B78] Li J. Liu J. Ren Y. Yang J. Liu P. (2014). Common chromosomal fragile site gene WWOX in metabolic disorders and tumors. *Int. J. Biol. Sci.* 10 142–148. 10.7150/ijbs.7727 24520212 PMC3920169

[B79] Li M. Y. Lai F. J. Hsu L. J. Lo C. P. Cheng C. L. Lin S. R. (2009). Dramatic co-activation of WWOX/WOX1 with CREB and NF-kappaB in delayed loss of small dorsal root ganglion neurons upon sciatic nerve transection in rats. *PLoS One* 4:e7820. 10.1371/journal.pone.0007820 19918364 PMC2771921

[B80] Lin H. P. Chang J. Y. Lin S. R. Lee M. H. Huang S. S. Hsu L. J. (2011). Identification of an in vivo MEK/WOX1 complex as a master switch for apoptosis in T cell leukemia. *Genes Cancer* 2 550–562. 10.1177/1947601911418498 21901168 PMC3161421

[B81] Llorens-Martin M. Jurado J. Hernandez F. Avila J. (2014). GSK-3β, a pivotal kinase in Alzheimer disease. *Front. Mol. Neurosci.* 7:46 10.3389/fnmol.2014.00046 PMC4033045 24904272

[B82] Lo C. P. Hsu L. J. Li M. Y. Hsu S. Y. Chuang J. I. Tsai M. S. (2008). MPP+-induced neuronal death in rats involves tyrosine 33 phosphorylation of WW domain-containing oxidoreductase WOX1. *Eur. J. Neurosci.* 27 1634–1646. 10.1111/j.1460-9568.2008.06139.x 18371080

[B83] Ludes-Meyers J. H. Kil H. Bednarek A. K. Drake J. Bedford M. T. Aldaz C. M. (2004). WWOX binds the specific proline-rich ligand PPXY: identification of candidate interacting proteins. *Oncogene* 23 5049–5055. 10.1038/sj.onc.1207680 15064722 PMC4143251

[B84] Ludes-Meyers J. H. Kil H. Parker-Thornburg J. Kusewitt D. F. Bedford M. T. Aldaz C. M. (2009). Generation and characterization of mice carrying a conditional allele of the Wwox tumor suppressor gene. *PLoS One* 4:e7775. 10.1371/journal.pone.0007775 19936220 PMC2777388

[B85] Mannella P. Brinton R. D. (2006). Estrogen receptor protein interaction with phosphatidylinositol 3-kinase leads to activation of phosphorylated Akt and extracellular signal-regulated kinase 1/2 in the same population of cortical neurons: a unified mechanism of estrogen action. *J. Neurosci.* 26 9439–9447. 10.1523/JNEUROSCI.1443-06.2006 16971528 PMC6674594

[B86] Martin L. J. (2012). Biology of mitochondria in neurodegenerative diseases. *Prog. Mol. Biol. Transl. Sci.* 107 355–415. 10.1016/B978-0-12-385883-2.00005-9 22482456 PMC3530202

[B87] McDonald C. B. Buffa L. Bar-Mag T. Salah Z. Bhat V. Mikles D. C. (2012). Biophysical basis of the binding of WWOX tumor suppressor to WBP1 and WBP2 adaptors. *J. Mol. Biol.* 422 58–74. 10.1016/j.jmb.2012.05.015 22634283 PMC3412936

[B88] Mohamoud H. S. Ahmed S. Jelani M. Alrayes N. Childs K. Vadgama N. (2018). A missense mutation in TRAPPC6A leads to build-up of the protein, in patients with a neurodevelopmental syndrome and dysmorphic features. *Sci. Rep.* 8:2053. 10.1038/s41598-018-20658-w 29391579 PMC5794855

[B89] Muñoz-Mayorga D. Guerra-Araiza C. Torner L. Morales T. (2018). Tau phosphorylation in female neurodegeneration: role of estrogens, progesterone, and prolactin. *Front. Endocrinol.* 9:133. 10.3389/fendo.2018.00133 29643836 PMC5882780

[B90] Niccoli T. Partridge L. (2012). Ageing as a risk factor for disease. *Curr. Biol.* 22 R741–R752. 10.1016/j.cub.2012.07.024 22975005

[B91] Nunez M. I. Ludes-Meyers J. Abba M. C. Kil H. Abbey N. W. Page R. E. (2005). Frequent loss of WWOX expression in breast cancer: correlation with estrogen receptor status. *Breast Cancer Res. Treat.* 89 99–105. 10.1007/s10549-004-1474-x 15692750 PMC4145848

[B92] O’Keefe L. V. Colella A. Dayan S. Chen Q. Choo A. Jacob R. (2011). Drosophila orthologue of WWOX, the chromosomal fragile site FRA16D tumour suppressor gene, functions in aerobic metabolism and regulates reactive oxygen species. *Hum. Mol. Genet.* 20 497–509. 10.1093/hmg/ddq495 21075834 PMC3016910

[B93] O’Leary V. B. Smida J. Buske F. A. Carrascosa L. G. Azimzadeh O. Maugg D. (2017). PARTICLE triplexes cluster in the tumor suppressor WWOX and may extend throughout the human genome. *Sci. Rep.* 7:7163. 10.1038/s41598-017-07295-5 28769061 PMC5541130

[B94] Perez-Alvarez M. J. Maza Mdel C. Anton M. Ordonez L. Wandosell F. (2012). Post-ischemic estradiol treatment reduced glial response and triggers distinct cortical and hippocampal signaling in a rat model of cerebral ischemia. *J. Neuroinflammation* 9:157. 10.1186/1742-2094-9-157 22747981 PMC3414748

[B95] Pierrat B. Simonen M. Cueto M. Mestan J. Ferrigno P. Heim J. (2001). SH3GLB, a new endophilin-related protein family featuring an SH3 domain. *Genomics* 71 222–234. 10.1006/geno.2000.6378 11161816

[B96] Quesada A. Lee B. Y. Micevych P. E. (2008). PI3 kinase/Akt activation mediates estrogen and IGF-1 nigral DA neuronal neuroprotection against a unilateral rat model of Parkinson’s disease. *Dev. Neurobiol.* 68 632–644. 10.1002/dneu.20609 18278798 PMC2667142

[B97] Ramírez C. M. Gonzalez M. Diaz M. Alonso R. Ferrer I. Santpere G. (2009). VDAC and ERalpha interaction in caveolae from human cortex is altered in Alzheimer’s disease. *Mol. Cell. Neurosci.* 42 172–183. 10.1016/j.mcn.2009.07.001 19595769

[B98] Reuven N. Shanzer M. Shaul Y. (2015). Tyrosine phosphorylation of WW proteins. *Exp. Biol. Med.* 240 375–382. 10.1177/1535370214565991 25627656 PMC4935225

[B99] Richter-Landsberg C. Leyk J. (2013). Inclusion body formation, macroautophagy, and the role of HDAC6 in neurodegeneration. *Acta Neuropathol.* 126 793–807. 10.1007/s00401-013-1158-x 23912309

[B100] Ried K. Finnis M. Hobson L. Mangelsdorf M. Dayan S. Nancarrow J. K. (2000). Common chromosomal fragile site FRA16D sequence: identification of the FOR gene spanning FRA16D and homozygous deletions and translocation breakpoints in cancer cells. *Hum. Mol. Genet.* 9 1651–1663. 10.1093/hmg/9.11.1651 10861292

[B101] Ross C. A. Poirier M. A. (2005). Opinion: what is the role of protein aggregation in neurodegeneration? *Nat. Rev. Mol. Cell Biol.* 6 891–898. 10.1038/nrm1742 16167052

[B102] Ruiz-Palmero I. Hernando M. Garcia-Segura L. M. Arevalo M. A. (2013). G protein-coupled estrogen receptor is required for the neuritogenic mechanism of 17beta-estradiol in developing hippocampal neurons. *Mol. Cell. Endocrinol.* 372 105–115. 10.1016/j.mce.2013.03.018 23545157

[B103] Ruiz-Riquelme A. Lau H. H. C. Stuart E. Goczi A. N. Wang Z. Schmitt-Ulms G. (2018). Prion-like propagation of β-amyloid aggregates in the absence of APP overexpression. *Acta Neuropathol. Commun.* 6:26. 10.1186/s40478-018-0529-x 29615128 PMC5883524

[B104] Sáez M. E. Gonzalez-Perez A. Martinez-Larrad M. T. Gayan J. Real L. M. Serrano-Rios M. (2010). WWOX gene is associated with HDL cholesterol and triglyceride levels. *BMC Med. Genet.* 11:148 10.1186/1471-2350-11-r148 PMC2967537 20942981

[B105] Safe S. Kim K. (2008). Non-classical genomic estrogen receptor (ER)/specificity protein and ER/activating protein-1 signaling pathways. *J. Mol. Endocrinol.* 41 263–275. 10.1677/JME-08-0103 18772268 PMC2582054

[B106] Salah Z. Aqeilan R. Huebner K. (2010). WWOX gene and gene product: tumor suppression through specific protein interactions. *Future Oncol.* 6 249–259. 10.2217/fon.09.152 20146584 PMC2832309

[B107] Savage M. J. Lin Y. G. Ciallella J. R. Flood D. G. Scott R. W. (2002). Activation of c-Jun N-terminal kinase and p38 in an Alzheimer’s disease model is associated with amyloid deposition. *J. Neurosci.* 22 3376–3385. 10.1523/JNEUROSCI.22-09-03376.2002 11978814 PMC6758401

[B108] Schultz L. Khera S. Sleve D. Heath J. Chang N. S. (2004). TIAF1 and p53 functionally interact in mediating apoptosis and silencing of TIAF1 abolishes nuclear translocation of serine 15-phosphorylated p53. *DNA Cell Biol.* 23 67–74. 10.1089/104454904322745943 14965474

[B109] Shah K. Lahiri D. K. (2015). A Tale of the Good and Bad: remodeling of the Microtubule Network in the Brain by Cdk5. *Mol. Neurobiol.* 54 2255–2268. 10.1007/s12035-016-9792-r7 26944284 PMC5011452

[B110] Shang Y. Hu X. DiRenzo J. Lazar M. A. Brown M. (2000). Cofactor dynamics and sufficiency in estrogen receptor-regulated transcription. *Cell* 103 843–852. 10.1016/S0092-8674(00)00188-4 11136970

[B111] Su W. P. Chen S. H. Chen S. J. Chou P. Y. Huang C. C. Chang N. S. (2012). “WW Domain-containing oxidoreductase is a potential receptor for sex steroid hormones,” in *Sex Hormones* ed. Raghvendra D. (Den Haag: InTech) 333–351. 10.5772/26043

[B112] Suzuki H. Katayama K. Takenaka M. Amakasu K. Saito K. Suzuki K. (2009). A spontaneous mutation of the Wwox gene and audiogenic seizures in rats with lethal dwarfism and epilepsy. *Genes Brain Behav.* 8 650–660. 10.1111/j.1601-183X.2009.00502.x 19500159

[B113] Sze C. I. Kuo Y. M. Hsu L. J. Fu T. F. Chiang M. F. Chang J. Y. (2015). A cascade of protein aggregation bombards mitochondria for neurodegeneration and apoptosis under WWOX deficiency. *Cell Death Dis.* 6:e1881. 10.1038/cddis.2015.251 26355344 PMC4650446

[B114] Sze C. I. Su M. Pugazhenthi S. Jambal P. Hsu L. J. Heath J. (2004). Down-regulation of WW domain-containing oxidoreductase induces Tau phosphorylation in vitro. A potential role in Alzheimer’s disease. *J. Biol. Chem.* 279 30498–30506. 10.1074/jbc.M401399200 15126504

[B115] Tabarki B. Al Mutairi F. Al Hashem A. (2015). The fragile site WWOX gene and the developing brain. *Exp. Biol. Med.* 240 400–402. 10.1177/1535370214561952 25416187 PMC4935222

[B116] Tang H. Zhang Q. Yang L. Dong Y. Khan M. Yang F. (2014). GPR30 mediates estrogen rapid signaling and neuroprotection. *Mol. Cell. Endocrinol.* 387 52–58. 10.1016/j.mce.2014.01.024 24594140 PMC4019970

[B117] Taylor R. C. Dillin A. (2013). XBP-1 is a cell-nonautonomous regulator of stress resistance and longevity. *Cell* 153 1435–1447. 10.1016/j.cell.2013.05.042 23791175 PMC4771415

[B118] Teyler T. J. Vardaris R. M. Lewis D. Rawitch A. B. (1980). Gonadal steroids: effects on excitability of hippocampal pyramidal cells. *Science* 209 1017–1018. 10.1126/science.7190730 7190730

[B119] Ueberham U. Ueberham E. Bruckner M. K. Seeger G. Gartner U. Gruschka H. (2005). Inducible neuronal expression of transgenic TGF-beta1 in vivo: dissection of short-term and long-term effects. *Eur. J. Neurosci.* 22 50–64. 10.1111/j.1460-9568.2005.04189.x 16029195

[B120] Valduga M. Philippe C. Lambert L. Bach-Segura P. Schmitt E. Masutti J. P. (2015). WWOX and severe autosomal recessive epileptic encephalopathy: first case in the prenatal period. *J. Hum. Genet.* 60 267–271. 10.1038/jhg.2015.17 25716914

[B121] van der Leij J. van den Berg A. Albrecht E. W. Blokzijl T. Roozendaal R. Gouw A. S. (2003). High expression of TIAF-1 in chronic kidney and liver allograft rejection and in activated T-helper cells. *Transplantation* 75 2076–2082. 10.1097/01.TP.0000069829.71088.88 12829915

[B122] Wang H. Y. Juo L. I. Lin Y. T. Hsiao M. Lin J. T. Tsai C. H. (2012). WW domain-containing oxidoreductase promotes neuronal differentiation via negative regulation of glycogen synthase kinase 3β. *Cell Death Differ.* 19 1049–1059. 10.1038/cdd.2011.188 22193544 PMC3354054

[B123] Warren S. G. Humphreys A. G. Juraska J. M. Greenough W. T. (1995). LTP varies across the estrous cycle: enhanced synaptic plasticity in proestrus rats. *Brain Res.* 703 26–30. 10.1016/0006-8993(95)01059-9 8719612

[B124] Yang H. C. Liang Y. J. Chen J. W. Chiang K. M. Chung C. M. Ho H. Y. (2012). Identification of IGF1, SLC4A4, WWOX, and SFMBT1 as hypertension susceptibility genes in Han Chinese with a genome-wide gene-based association study. *PLoS One* 7:e32907. 10.1371/journal.pone.0032907 22479346 PMC3315540

[B125] Yao M. Nguyen T. V. Pike C. J. (2007). Estrogen regulates Bcl-w and Bim expression: role in protection against beta-amyloid peptide-induced neuronal death. *J. Neurosci.* 27 1422–1433. 10.1523/JNEUROSCI.2382-06.2007 17287517 PMC6673600

[B126] Zeng L. Zielinska H. A. Arshad A. Shield J. P. Bahl A. Holly J. M. (2016). Hyperglycaemia-induced chemoresistance in breast cancer cells: role of the estrogen receptor. *Endocr. Relat. Cancer* 23 125–134. 10.1530/ERC-15-0507 26647383

[B127] Zhang Q. G. Wang R. Khan M. Mahesh V. Brann D. W. (2008). Role of Dickkopf-1, an antagonist of the Wnt/β-catenin signaling pathway, in estrogen-induced neuroprotection and attenuation of tau phosphorylation. *J. Neurosci.* 28 8430–8441. 10.1523/JNEUROSCI.2752-08.2008 18716201 PMC2639789

